# Polo-Like Kinase 2-Dependent Phosphorylation of NPM/B23 on Serine 4 Triggers Centriole Duplication

**DOI:** 10.1371/journal.pone.0009849

**Published:** 2010-03-24

**Authors:** Annekatrin Krause, Ingrid Hoffmann

**Affiliations:** Cell Cycle Control and Carcinogenesis, German Cancer Research Center (DKFZ), Heidelberg, Germany; Duke University Medical Centre, United States of America

## Abstract

Duplication of the centrosome is well controlled during faithful cell division while deregulation of this process leads to supernumary centrosomes, chromosome missegregation and aneuploidy, a hallmark of many cancer cells. We previously reported that Polo-like kinase 2 (Plk2) is activated near the G1/S phase transition, and regulates the reproduction of centrosomes. In search for Plk2 interacting proteins we have identified NPM/B23 (Nucleophosmin) as a novel Plk2 binding partner. We find that Plk2 and NPM/B23 interact *in vitro* in a Polo-box dependent manner. An association between both proteins was also observed *in vivo*. Moreover, we show that Plk2 phosphorylates NPM/B23 on serine 4 *in vivo* in S-phase. Notably, expression of a non-phosphorylatable NPM/B23 S4A mutant interferes with centriole reduplication in S-phase arrested cells and leads to a dilution of centriole numbers in unperturbed U2OS cells. The corresponding phospho-mimicking mutants have the opposite effect and their expression leads to the accumulation of centrioles. These findings suggest that NPM/B23 is a direct target of Plk2 in the regulation of centriole duplication and that phosphorylation on serine 4 can trigger this process.

## Introduction

Centrosomes play fundamental roles in regulating chromosome segregation in mitosis and the interphase microtubule cytoskeleton. The centrosome consists of a pair of centrioles surrounded by amorphous pericentriolar material. Like chromosomes, the centrioles duplicate only once per cell cycle. Centriole duplication is initiated at the G1/S boundary and is completed in S-phase of the cell cycle, which coincides with DNA replication [1]. Interestingly, there is a correlation between excess centrosomes, aneuploidy, and cancer [2]. Errors in centriole duplication lead to cells with too many or too few centrosomes; the resulting monopolar spindles or bipolar spindles, which harbor clustered centrosomes at their spindle poles, disrupt chromosome segregation and can lead to genomic instability [3]. Therefore, extra centrosomes alone are sufficient to promote chromosome missegregation during bipolar cell division. Thus, understanding regulatory mechanisms governing centrosome duplication provides insights into both normal cell behavior and tumorigenesis.

Several human protein kinases were shown to be critical for centriole duplication and assembly. The duplication of the centrosome is initiated by the disengagement of the two centrioles at the end of mitosis [4]. This is followed by an activation of Polo-like kinase 4 (Plk4) and the subsequent recruitment of hSAS-6 and other centriolar components to maternal centrioles, which are critical steps in procentriole assembly [5]. In addition, Plk2 is localized to centrosomes and its kinase activity is also required for centriole duplication [6] [7]. Plk2 substrates in this process are currently unknown. Plk2 and Plk4 exhibit distinct structural properties especially at the Polo-box domains (PBD) and have their own substrate specificities consequently [8] [9]. Cdk2 is also necessary for initiation of centriole duplication [10]. A few candidate substrates were identified as being responsible for Cdk2 function in centrosome duplication. For example, Cdk2 phosphorylates NPM/B23 (Nucleophosmin) [11] on threonine (T) 199 resulting in its dissociation from the centrosome prior to the initiation of centriole duplication [12]. These results suggest that NPM/B23 may negatively regulate this process. NPM/B23 reassociates with centrosomes during mitosis [11] [13]. Phosphoprotein profiling of M-phase arrested cells by nocodazole treatment also shows that NPM/B23 is phosphorylated in mitosis on serine (S) 4 which is dependent on Polo-like kinase 1 (Plk1) [14].

NPM/B23 is a multifunctional protein that is involved in a number of cellular activities. It has been related to both growth-suppressive and proliferative roles in the cell. A large fraction of NPM/B23 resides in the nucleoli, although it shuttles rapidly between the nucleus and the cytoplasm [15]. On the one hand, NPM/B23 is frequently overexpressed in solid tumors of diverse histological origin, whereas on the other hand, the *NPM1* locus is involved in chromosomal translocations or deleted in various kinds of haematological malignancies and solid tumors [16]. *NPM1* is essential for embryonic development and the maintenance of genomic stability in mice. Interestingly, *NPM1* inactivation leads to unrestricted centrosome duplication, and supernumary centrosomes were observed [17].

To find substrates of Plk2 kinase in centrosome duplication we used a biochemical approach and identified NPM/B23. We show that Plk2 interacts with and phosphorylates NPM/B23 on S4 in both mitosis and S-phase while Plk1 seems to be involved only in its mitotic phosphorylation. Furthermore, we demonstrate that Plk2-dependent phosphorylation of NPM/B23 on S4 triggers both centriole reduplication and normal centrosome reproduction. Our results suggest that Plk2 exerts its role in centrosome duplication by phosphorylating NPM/B23 on S4.

## Results

### Identification of NPM/B23 as a novel Plk2 binding partner

**Figure 1:**
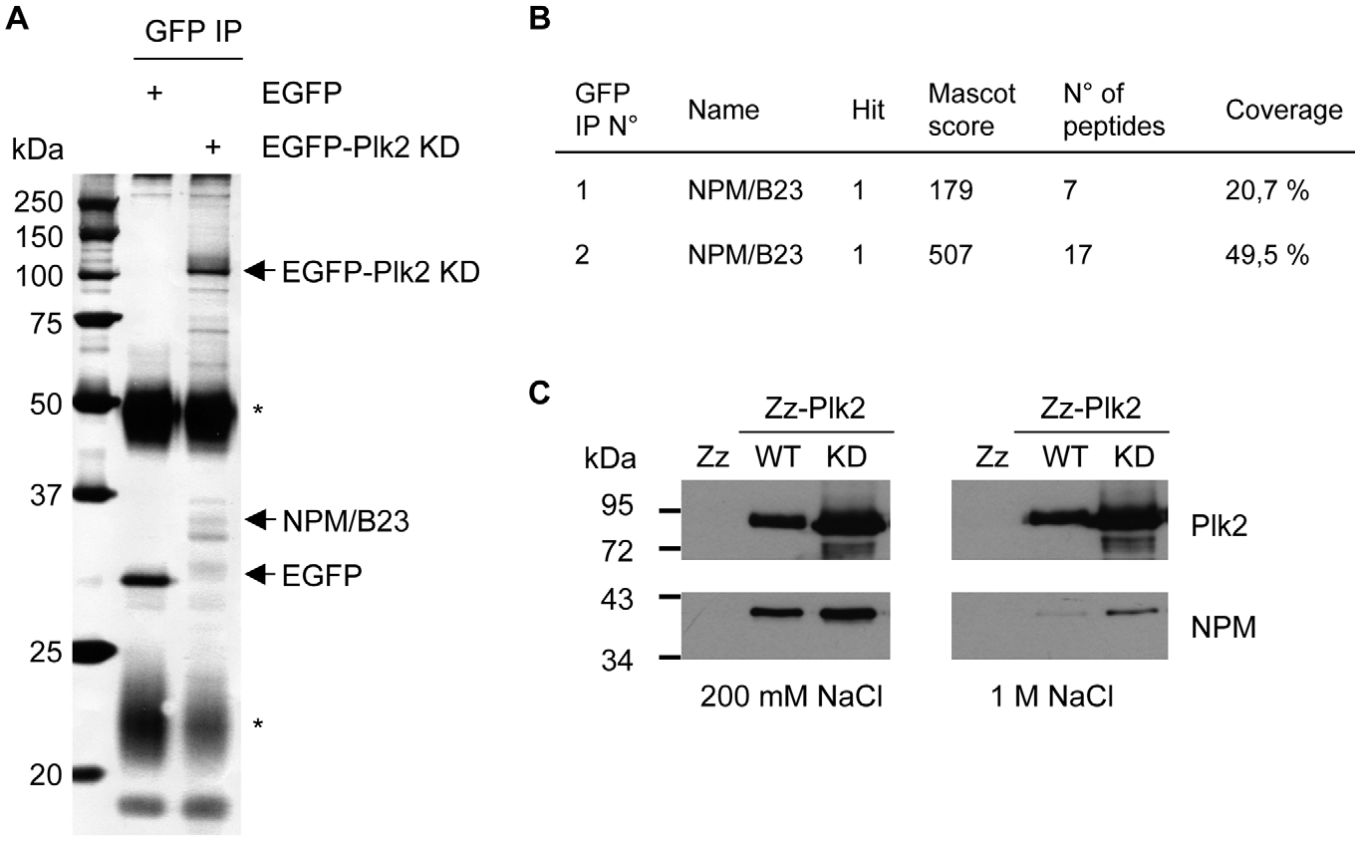
Identification of NPM/B23 as Plk2 interacting partner. **A** EGFP-tagged Plk2 kinase dead (KD, N210A) or EGFP alone, overexpressed in HeLa cells, was immunoprecipitated by anti-GFP antibodies and immunocomplexes were resolved by SDS-PAGE. Co-immunoprecipitating proteins were detected by silver staining. The co-precipitating protein NPM/B23 was identified by mass spectrometry analysis of a protein band with an approximate size of 35 kDa. IgG bands are marked with asterisks (*). **B** Mass spectrometry analysis results of two independent EGFP-Plk2 KD immunoprecipitations where NPM/B23 was identified as a Plk2 interaction partner. **C** Zz-Plk2 wildtype (WT), KD or Zz alone as control were bound to IgG sepharose and incubated with HeLa lysate. After washing protein complexes, interacting proteins were eluted under low (200 mM NaCl) or high salt (1 M NaCl) conditions and analyzed by SDS-PAGE and Western blotting using anti-Plk2 and anti-NPM/B23 antibodies.

**Figure 2:**
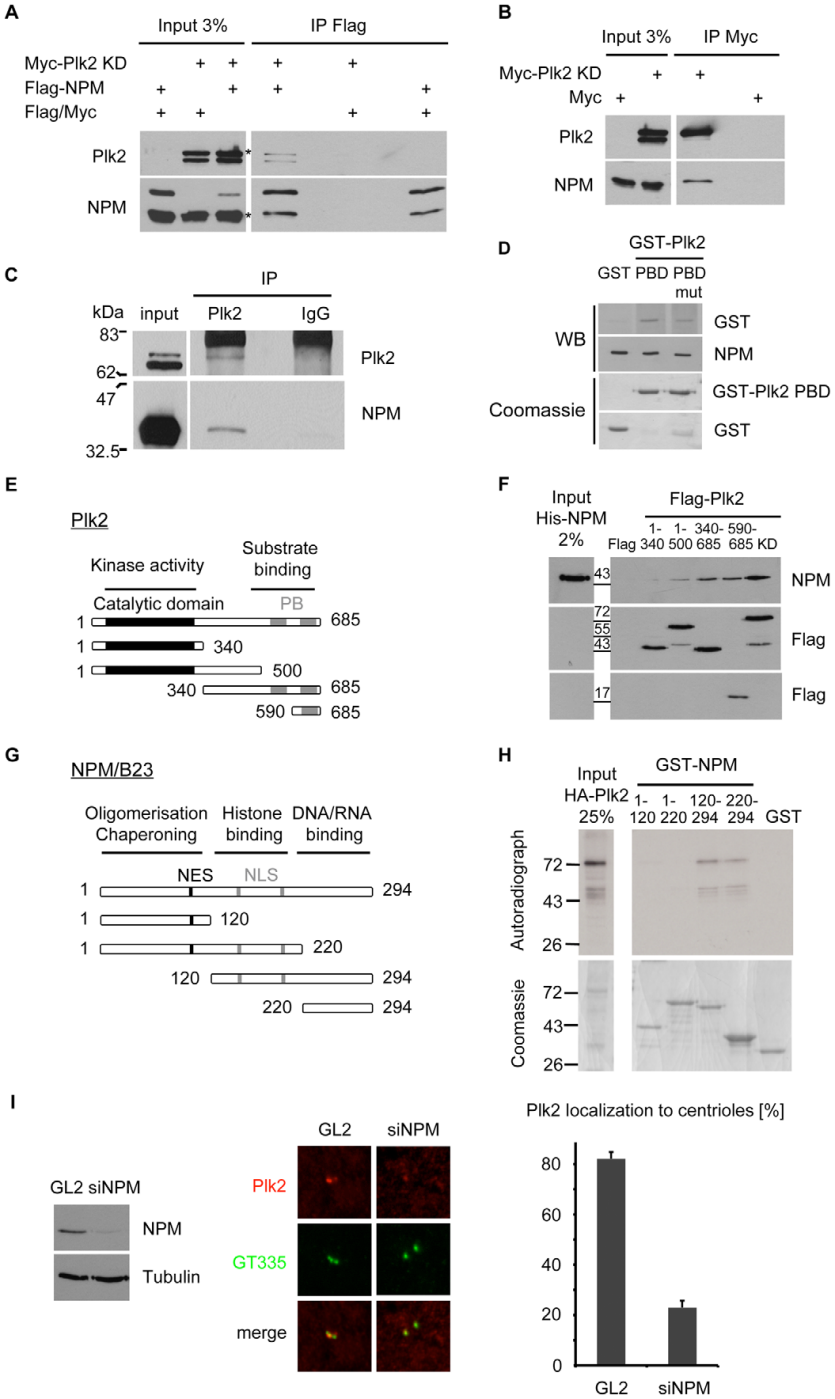
Plk2 interacts with NPM/B23 *in vivo* and *in vitro*. **A** After overexpression of Myc-Plk2 KD and Flag-NPM/B23 in 293T cells, cell extracts were subjected to immunoprecipitations using anti-Flag antibodies. Co-precipitation of Myc-Plk2 KD by Flag-NPM/B23 was detected by Western blotting using anti-Plk2 and anti-NPM/B23 antibodies. Asterisks (*), endogenous NPM/B23. **B** Myc-Plk2 KD was overexpressed in 293T cells and immunoprecipitated with anti-Myc antibodies. Complex formation of Myc-Plk2 KD and endogenous NPM/B23 was analyzed by Western blot with anti-Plk2 and anti-NPM/B23 antibodies. **C** Endogenous Plk2 was immunoprecipitated from HeLa lysates by specific antibodies. The interaction was verified by Western blotting using anti-NPM/B23 and anti-Plk2 antibodies. IgG, random antibody control. **D** Far Western blot analysis. Recombinant His-tagged NPM/B23 was resolved by SDS-PAGE, blotted onto nitrocellulose and incubated with GST-Plk2 PBD, -PBD mutant or GST (control) to allow interaction. Protein bound to NPM/B23 was detected with anti-GST antibodies. Equal amounts of GST-PBD, -PBD mutant and GST used for ligand binding are demonstrated by Coomassie blue staining. **E** Scheme of Plk2 truncated versions. **F** Flag-Plk2 truncated versions (E) were overexpressed in 293T cells, immunoprecipitated by anti-Flag antibodies and incubated with recombinant His-NPM/B23 in *in vitro* binding assays. Complexes were separated by SDS-PAGE and analyzed by Western blot using anti-NPM/B23 and anti-Flag antibodies. **G** Scheme of NPM/B23 truncated versions. **H** Glutathione sepharose-bound GST-NPM/B23 truncated versions (G) or GST (control) were incubated with *in vitro* translated, [^35^S]-methionine-labeled HA-Plk2 in pull down assays. Complexes were resolved by SDS-PAGE. After Coomassie blue staining, interactions were detected by autoradiography. **I** NPM/B23 was downregulated by siRNA in U2OS cells as demonstrated in a Western blot analysis (left panel). Indirect immunofluorescence analysis was performed for Plk2 (red) and centriolar GT335 (green) (middle panel). Localization of centriolar Plk2 was quantified in 3 independent experiments with >150 analyzed cells each (right panel). GL2, siRNA control; loading control, α-Tubulin.

To identify Plk2 interacting proteins and potential substrates, we transfected an EGFP-tagged Plk2 kinase-inactive mutant (Plk2 KD) into HeLa cells and immunoprecipitated the proteins by using anti-GFP antibodies. We used a kinase-inactive mutant since it stabilizes the interaction with substrates. Several interacting bands could be identified that were absent in the control immunoprecipitation (Fig. 1A). These bands were subjected to mass spectrometry analysis where a band, migrating at 35 kDa, was identified as NPM/B23 (Nucleophosmin) (Fig. 1B). The interaction was verified in a pulldown assay using purified recombinant Zz-tagged kinase active Plk2 (Plk2 WT) or KD bound to IgG sepharose that was incubated with a HeLa cell lysate. Interacting proteins were eluted under low and high salt (NaCl) conditions and then subjected to Western blot analysis. Fig. 1C shows that NPM/B23 bound to both Plk2 WT and Plk2 KD. We further tested the interaction between NPM/B23 and Plk2 by co-immunoprecipitation assays using either co-transfection of Flag-NPM/B23 and Myc-Plk2 KD or transfection of Myc-Plk2 KD alone into 293T cells. Anti-Flag antibodies co-immunoprecipitated Plk2 (Fig. 2A) and anti-Myc antibodies co-immunoprecipitated endogenous NPM/B23 (Fig. 2B) demonstrating the physical interaction between these two proteins. To verify that Plk2 and NPM/B23 interact *in vivo* Plk2 was immunoprecipitated from HeLa cell lysates with specific antibodies [6]. Using NPM/B23 antibodies in the following immunoblot we detected an interaction between endogenous proteins *in vivo* (Fig. 2C).

As the conserved Polo-boxes of most Plks are required for their function and interaction with substrates [18], we asked the question if binding between NPM/B23 and Plk2 is mediated through the Polo-box domain (PBD) of Plk2. We used a Far Western ligand binding assay to test the ability of the PBD to bind NPM/B23. The assay was carried out using recombinant NPM/B23 with either GST, GST-PBD, or GST-PBD-mutant [7]. Ligand-binding was analyzed with anti-GST antibodies. Fig. 2D shows that GST-PBD bound to NPM/B23 while no binding was observed when GST-alone was incubated with NPM/B23. In addition, reduced binding was observed in comparison to the wildtype when GST-PBD-mutant was used. These results suggest that the PBD domain of Plk2 is required for the binding to NPM/B23. Next, to identify the essential region of Plk2 required for the interaction with NPM/B23, we generated Flag-tagged Plk2 truncated versions (Fig. 2E) that were ectopically expressed in 293T cells, and immunoprecipitated with Flag-antibodies. Immunoprecipitates were then incubated with purified recombinant His-tagged NPM/B23. As clearly shown in Fig. 2F Flag-Plk2-(amino acids (aa) 340–685) and Flag-Plk2-(aa 590–685) efficiently bound to NPM/B23 implying that the region between amino acid residues 340 and 585 of Plk2, harboring the C-terminal regulatory part of the kinase, is important for the interaction with NPM/B23. Moreover, since Flag-Plk2-(aa 590–685), comprising only the second Polo-box, bound NPM/B23 as efficiently as the longer Flag-Plk2-(aa 340–685) fragment, the second Polo-box of Plk2 seems to be sufficient for the interaction with NPM/B23. Since Plk2 binds to unphosphorylated, bacterially produced NPM/B23 in these experiments, a pre-phosphorylation event by an additional kinase might not be required for the PBD-dependent interaction between Plk2 and NPM/B23. However, a self-priming mechanism as described previously for Plk1 and PBIP1 [19] might strengthen the physical interaction between NPM/B23 and Plk2. Vice versa, to map the Plk2-interacting domain on NPM/B23 we constructed GST-tagged deletion mutants including NPM/B23-(aa 1–120), NPM/B23-(aa 1–220), NPM/B23-(aa 120–294) and NPM/B23-(aa 220–294) (Fig. 2G). We then tested the interaction between Plk2 and each of these NPM/B23 deletion mutants. A GST-pulldown assay was used to identify the domain of NPM/B23 necessary for binding to *in vitro* translated [^35^S]-HA-Plk2. We found that NPM/B23-(aa 120–294) and NPM/B23-(aa 220–294) retained the ability to bind to Plk2, whereas NPM/B23-(aa 1–120) and NPM/B23-(aa 1–220) did not. These results indicate that NPM/B23 physically interacts with Plk2 through its amino acid residues 220–294 (Fig. 2H). Since we have shown that Plk2 interacts with NPM/B23 through its PBD (Fig. 2D), we asked whether the localization of Plk2 at the centrosome would be impaired when NPM/B23 is absent. We find that down-regulation of NPM/B23 by siRNAs to ∼20% but not control siRNAs (Fig. 2I, left) leads to marked loss of the Plk2 signal at the centrosomes (Fig. 2I, middle and right) which suggests that NPM/B23 recruits Plk2 to the centrosome most likely through binding to the PBD of Plk2.

### Plk2 directly phosphorylates serine 4 on NPM/B23

**Figure 3:**
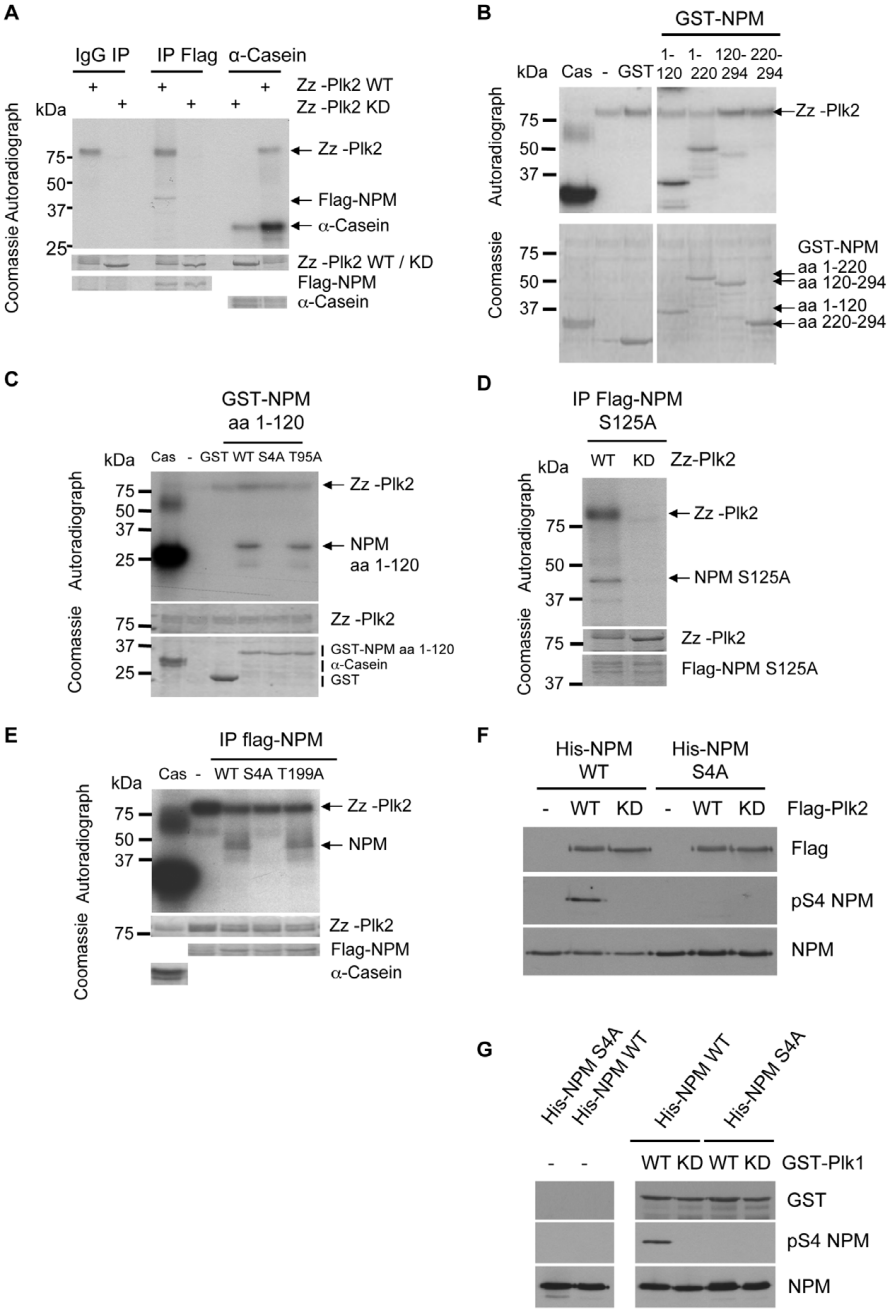
Plk2 phosphorylates NPM/B23 on serine 4. **A** Flag-NPM/B23 was overexpressed in 293T cells. After lysis, immunoprecipitations with anti-Flag antibodies or random IgGs as control were performed. Immunoprecipitated Flag-NPM/B23 and controls were subjected to *in vitro* kinase assays with Zz-Plk2 WT or KD, purified from bacteria, in the presence of [γ^32^P]-ATP. To assess Zz-Plk2 WT and KD kinase activity, *in vitro* kinase assays, supplemented with [γ^32^P]-ATP, were performed using α-Casein as substrate. Kinase assays were resolved by SDS-PAGE. After Coomassie staining of the gel, phosphorylation was detected by autoradiography. **B** To identify the Plk2 phosphorylation site on NPM/B23, *in vitro* kinase assays were carried out using Zz-tagged Plk2 WT in combination with GST-tagged NPM/B23 fragments shown in Fig. 2G, as control GST alone, in the presence of [γ^32^P]-ATP. After SDS-PAGE, followed by Coomassie blue staining of the gel, analysis was done by autoradiography. **C** To verify the NPM/B23 phosphorylation site, NPM/B23 serine 4 and threonine 95 were mutated to alanine (A). GST-NPM/B23 (aa 1–120) WT, S4A and T95A were incubated with Zz-Plk2 in an *in vitro* kinase assay and analyzed as in (B). **D** *In vitro* kinase assay using immunoprecipitated Flag-NPM/B23 S125A mutant from 293T cells together with Zz-Plk2 WT and Zz-Plk2 KD from bacteria. Analysis was done as in (A). **E** *In vitro* kinase assay, assembled and analyzed as in (A) besides using immunoprecipitated Flag-NPM/B23 WT, S4A or T199A as substrates. **F** *In vitro* kinase assay using immunoprecipitated Flag-Plk2 WT or KD from 293T cells together with His-tagged NPM/B23 WT or S4A from bacteria. Reactions were subjected to Western blot analysis using anti-pS4 NPM/B23 and anti-NPM/B23 antibodies. **G** *In vitro* kinase assay, assembled and analyzed as described in (F) besides using GST-tagged Plk1 WT or KD.

Furthermore, to address whether Plk2 could phosphorylate NPM/B23, we carried out *in vitro* kinase reactions. Flag-NPM/B23 was transfected into 293T cells, immunoprecipitated with Flag-antibodies and then incubated with Zz-Plk2 WT and Zz-Plk2 KD as control in the presence of [γ^32^P]-ATP. The reaction mixtures were separated by SDS–PAGE and subjected to autoradiography (Fig. 3A). The results showed that Plk2 phosphorylates NPM/B23 *in vitro*. To identify the Plk2 phosphorylation site on NPM/B23, we first determined the region in the NPM/B23-protein that is phosphorylated by Plk2. For this, NPM/B23 deletion mutants (Fig. 2G) were used in an *in vitro* kinase assay with active recombinant Zz-Plk2. Results in Fig. 3B reveal that *in vitro* phosphorylation occurs in the deletion mutants NPM/B23-(aa 1–120) and NPM/B23-(aa 1–220). A slight phosphorylation was also detected in NPM/B23-(aa 120–294) (Fig. 3B). To identify Plk2 specific phosphorylation sites we mutated several S and T amino acid residues within the amino acid region 1 to 125 in NPM/B23. These comprise S4 [14], T95 [15], T199 [12] and S125. We included the fragment containing S125 in our analysis since the region surrounding S125 ressembles a putative Plk2 phosphorylation site [20]. To show which sites are phosphorylated by Plk2, we first generated GST-tagged N-terminal (aa 1–120) fragments where S4 and T95 were mutated to alanine (A). Both purified fragments were subjected along with the WT fragment to *in vitro* kinase assays using active Zz-tagged Plk2 WT. While the fragment containing the T95A mutant was still phosphorylated to a similar extent as the WT fragment, no incorporation of phosphate was observed when S4 was mutated to alanine (Fig. 3C). Mutation of the putative Plk2 consensus site S125 to alanine still resulted in incorporation of [γ^32^P] into NPM/B23 suggesting that this site is not phosphorylated by Plk2 (Fig. 3D). Then we tested phosphorylation deficient mutants of full-length NPM/B23 WT, S4A and T199A, upon phosphorylation by Plk2. We expressed the constructs in 293T cells, immunoprecipitated the proteins with anti-Flag-antibodies and subjected the immunoprecipitates to an *in vitro* kinase assay with recombinant Zz-tagged Plk2. While [γ^32^P] was efficiently incorporated in both NPM/B23 WT and the T199A mutant, the NPM/B23 S4A mutant was not phosphorylated by Plk2 (Fig. 3E) confirming that S4 is the site that is phosphorylated by Plk2. To further verify that NPM/B23 is phosphorylated by Plk2 on S4 we used a phospho-specific antibody against NPM/B23 phosphorylated on S4 (pS4 NPM/B23) in a reaction where His-tagged NPM/B23 WT or NPM/B23 S4A was incubated with Plk2 WT or KD in an *in vitro* kinase assay. Fig. 3F shows that the NPM/B23 S4A-mutant cannot be phosphorylated by active Plk2. Similar to Plk2, Plk1 is also able to phosphorylate NPM/B23 on S4 (Fig. 3G and [14]). Together, these results show that NPM/B23 is phosphorylated on S4 by both Plk2 and Plk1 *in vitro*.

### Plk-dependent phosphorylation of NPM/B23 is cell cycle-regulated

**Figure 4:**
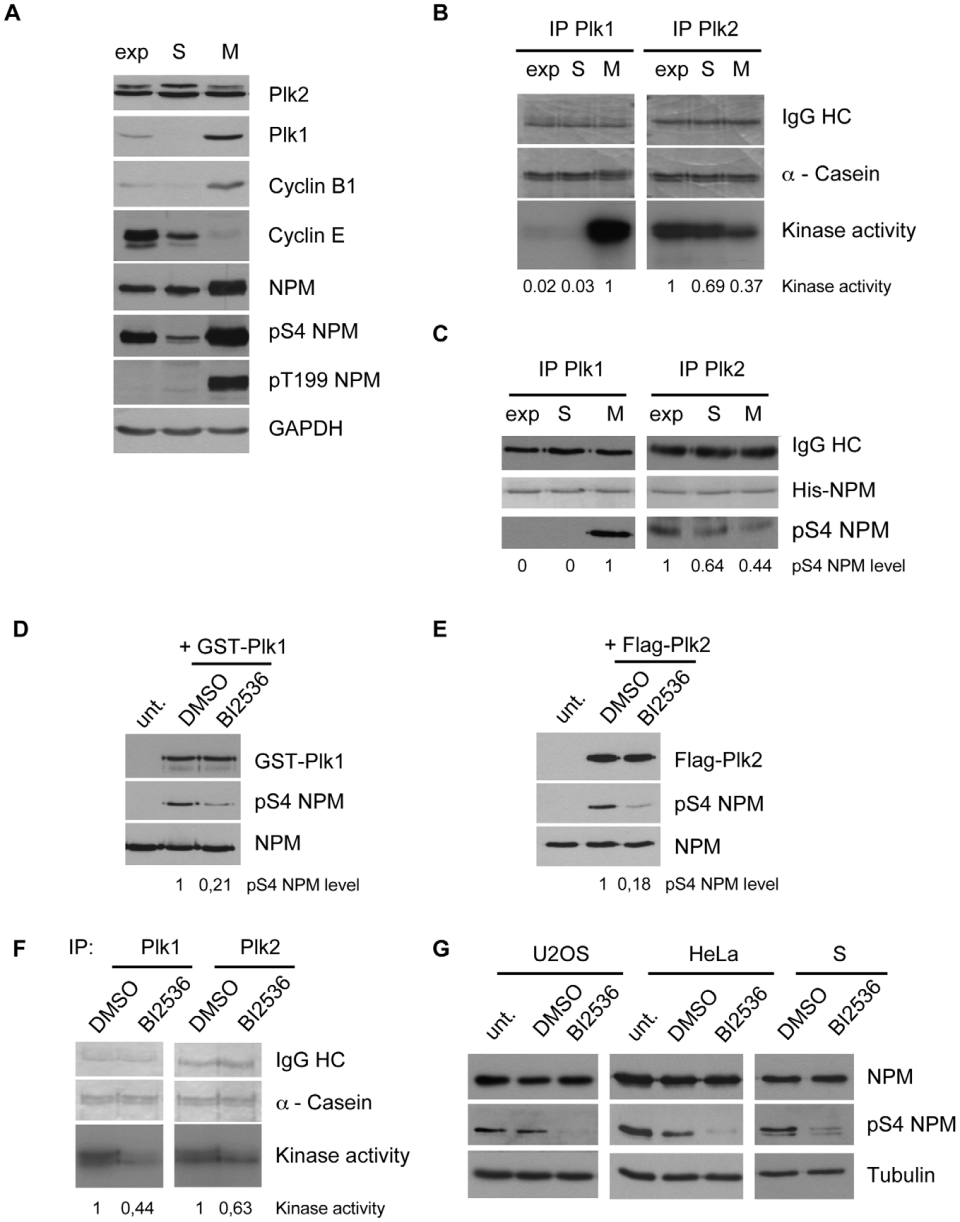
Phosphorylation of NPM/B23 serine 4 is cell cycle regulated. **A** Cell extracts from HeLa cells, asynchronously (exp) grown or synchronized in S-phase (S) by 4 mM hydroxyurea for 40 h or in M-phase (M) by 100 ng/ml nocodazole for 15 h, were examined by Western blotting using the indicated antibodies. GAPDH, loading control. **B** Plk1 or Plk2 were immunoprecipitated from HeLa extracts, either exponentially grown or arrested in M-phase or in S-phase as described in (A), and subjected to *in vitro* kinase assays using α-Casein as a substrate in the presence of [γ^32^P]-ATP. For experimental details see in Fig. 3A. HC, IgG heavy chain. **C** Plk1 and Plk2 were immunoprecipitated from HeLa extracts as described in (B) and subjected to *in vitro* kinase assays with His-tagged NPM/B23 as substrate. Western blotting was performed by NPM/B23 pS4 antibodies. Equal His-NPM/B23 levels are shown by Ponceau S staining. **D** *In vitro* kinase assays using GST-tagged Plk1 and His-NPM/B23 were performed in the presence of 100 mM BI2536 or DMSO. Phosphorylation of His-NPM/B23 was detected by Western blot with anti-pS4 NPM/B23 antibodies. Loading control, NPM/B23; unt., untreated. **E** *In vitro* kinase assays using immunoprecipitated Flag-Plk2 WT from 293T cells and His-NPM/B23 as substrate. The experiment was performed as described in (D). **F** HeLa cells were incubated with 100 nM BI2536 or DMSO for 2 h. Cell extracts were used for immunoprecipitations with anti-Plk1 or anti-Plk2 antibodies and subjected to *in vitro* kinase assays using α-Casein as substrate, supplemented with [γ^32^P]-ATP. Kinase assays were analyzed as described in (B). **G** Asynchronous HeLa or U2OS cells or S-phase arrested HeLa cells as in (A) were treated with 100 nM BI2536 for 2 h. Cell extracts were prepared for Western blotting with anti-NPM/B23 and anti-pS4 NPM/B23 antibodies. Loading control, α-Tubulin. Quantifications of kinase activities or protein levels in this figure were generated by ImageJ (NIH).

The activity profiles of Plk1 and Plk2 are different during the cell cycle. Plk1 is activated near the G2/M phase transition until the end of cytokinesis [21] while Plk2 is activated in G1 near the G1/S phase transition [6]. We therefore determined the phosphorylation profile of NPM/B23 on S4 during the cell cycle and compared its presence in HeLa cell extracts from S-phase and mitosis. While S4 was heavily phosphorylated in M-phase, a slight but distinct phosphorylation of this site also occurs in S-phase (Fig. 4A). We then compared the kinase activities of Plk1 and Plk2 that were immunoprecipitated from cells arrested in S-phase (hydroxyurea block) or pro-metaphase (nocodazole block) on α-Casein or His-NPM/B23 as substrate. While active Plk1 could only be immunoprecipitated from M-Phase, Plk2 immunoprecipitates were active in both M- and S-phases on both α-Casein (Fig. 4B) and His-tagged NPM/B23 (Fig. 4C) as substrates. Since Plk2 but not Plk1 is active in S-phase (Fig. 4B and 4C) [21] [6] the phosphorylation of NPM/B23 on S4 in S-phase accounts for a dependency on Plk2 activity at this cell cycle stage. To further demonstrate the involvement of both Plk1 and Plk2 in the regulation of NPM/B23 S4 phosphorylation we made use of the small molecule inhibitor, BI2536 that inhibits both Plk1 and Plk2 kinase activities [22]. We tested the effect of inhibiting Plk1 and Plk2 kinases by BI2536 in *in vitro* kinase assays using the pS4 NPM/B23 antibody. Both Plk1 (Fig. 4D) and Plk2 kinase activities (Fig. 4E) were inhibited in their abilities to phosphorylate NPM/B23 on S4 *in vitro*. To assess the inhibitory effect of BI2536 on Plk1 and Plk2 *in vivo*, HeLa cells were treated with BI2536 and Plk1 and Plk2 were immunoprecipitated by specific antibodies and subjected to a kinase assay with α-Casein as exogenous substrate. As shown in Fig. 4F, both Plk1 and Plk2 can be partially inhibited by BI2536 *in vivo*, but Plk1 was inhibited to a stronger extent. To analyze the effect of BI2536 on the NPM/B23 S4 phosphorylation, exponentially growing U2OS and HeLa cells were treated with BI2536 and analyzed consequently in a Western blot for pS4 NPM/B23 levels. In both cell lines, BI2536 treatment led to a decrease in pS4 NPM/B23 levels (Fig. 4G). Since Plk1 is not active in S-phase but Plk2 is, the effect of BI2536 on S4 phosphorylation of NPM/B23 was also investigated in S-phase arrested HeLa cells. As shown in Fig. 4G, BI2536 treatment also results in a decrease of pS4 NPM/B23 levels in S-phase cells. Taken together, phosphorylation of NPM/B23 S4 can be mediated by both Polo-like kinases, Plk1 and Plk2 (Fig. 4D–G). However, since both kinases display differential activities during the cell cycle (Fig. 4B and 4C), a phosphorylation of NPM/B23 by Plk1 is suggested for mitosis while a phosphorylation of NPM/B23 on S4 in S-phase is likely to be catalyzed by Plk2.

### Phosphorylation on S4 of NPM/B23 is dependent on Plk2 in S-phase

**Figure 5:**
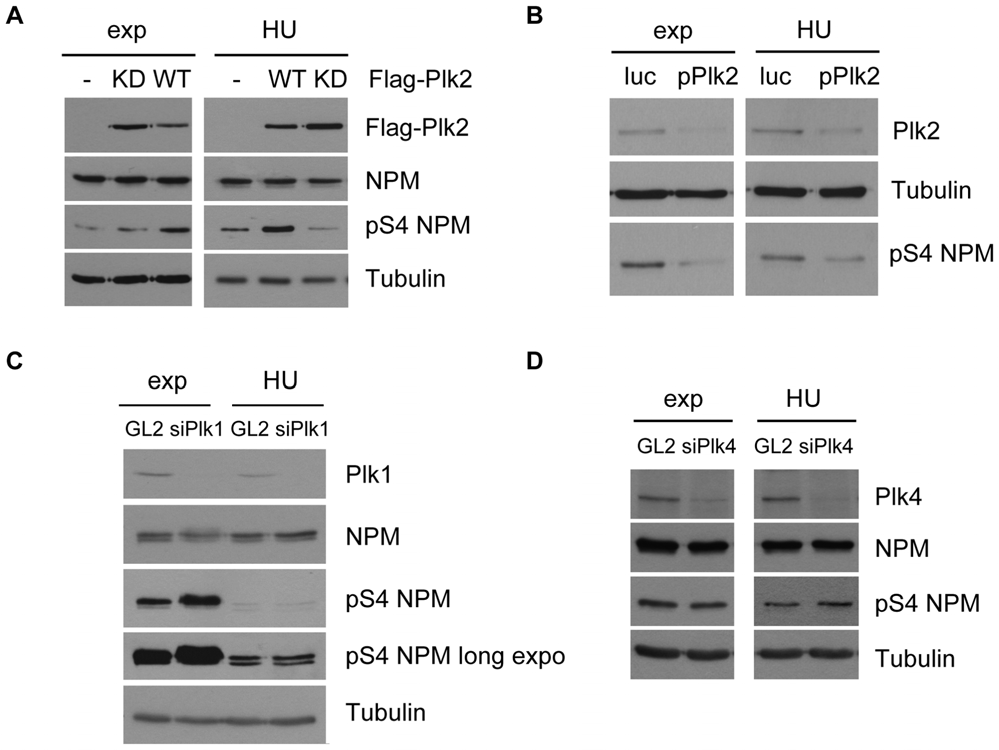
NPM/B23 serine 4 phosphorylation in S-phase is mediated by Plk2. **A** Flag-Plk2 WT, KD or Flag-alone were overexpressed in 293T cells. 8 h after transfections cells were arrested with 4 mM hydroxyurea (HU) for 40 h. Exponentially growing cells were harvested 48 h after transfections. Cell extracts were analyzed by SDS-PAGE and Western blot using anti-Flag, anti-NPM/B23, anti-pS4 NPM/B23 and anti-α-Tubulin (loading control) antibodies. **B** RNAi of Plk2 was mediated by overexpression of pSuper-RNAi-Plk2 and pSuper-RNAi-Luciferase as control in 293T cells. Cell cycle synchronization and analyses were carried out as in (A). To detect Plk2 downregulation in Western blot, membranes were probed with anti-Plk2 antibodies. **C** RNAi of Plk1 in HeLa cells was mediated by Lipofectamine2000-transfections of Plk1 specific siRNAs. GL2 siRNAs were used as control. Cell extracts were analyzed by Western blotting with the indicated antibodies. Loading control, α-Tubulin. **D** Plk4 was downregulated in U2OS cells by Lipofectamine2000 mediated siRNA transfections, as control GL2 was used. S-phase arrest was achieved as described in (A). Downregulation of Plk4 and pS4 NPM/B23 levels were examined by Western blot analysis with the indicated antibodies. Loading control, α-Tubulin.

To directly confirm that NPM/B23 is phosphorylated by Plk2 in S-phase HeLa cells were transfected with either Flag-tagged Plk2 WT or Plk2 KD and then treated with hydroxyurea (HU) to arrest cells in early S-phase. Western blot analysis shows that phosphorylation of NPM/B23 on S4 is increased upon Plk2 WT transfection while Plk2 KD expression decreased the signal in comparison to non-transfected control cells most likely by competing out the endogenous active Plk2 (Fig. 5A). To confirm these data we depleted Plk2 by RNAi using shRNA-treatment [6]. As shown in Fig. 5B the phosphorylated NPM/B23-S4 signal was reduced in both asynchronous and HU-treated 293T cells suggesting that Plk2 is regulating S4 phosphorylation *in vivo*.

Although Plk1 is not active in S-phase (Fig. 4B and 4C), low protein levels of Plk1 are detectable in this cell cycle stage (Fig. 5C). To rule out, that these residual amounts of Plk1 are responsible for a NPM/B23 S4 phosphorylation in S-phase, Plk1 was depleted from S-phase arrested HeLa cells by siRNA transfections and analyzed for pS4 NPM/B23 levels. Fig. 5C reveals that a downregulation of Plk1 in S-phase arrested cells did not affect pS4 NPM/B23 protein levels. This again shows that a S-phase linked phosphorylation of NPM/B23 S4 is likely to be carried out by Plk2.

Since besides Plk2 also Plk4 regulates centriole duplication at the G1/S-phase transition and in order to rule out that the NPM/B23-S4 phosphorylation is also dependent on Plk4 in early S-phase, we depleted Plk4 by RNAi from asynchronous and HU-treated U2OS cells. However, in neither case, Plk4 siRNA decreased the signal of NPM/B23-S4 phosphorylation (Fig. 5D) suggesting that Plk4 cannot phosphorylate NPM/B23 at this site. Taken together, our results point to a phosphorylation of NPM/B23 on S4 by Plk1 in mitosis, while Plk2 catalyzes this phosphorylation in S-phase.

### NPM/B23 phosphorylation on S4 triggers centriole duplication

**Figure 6:**
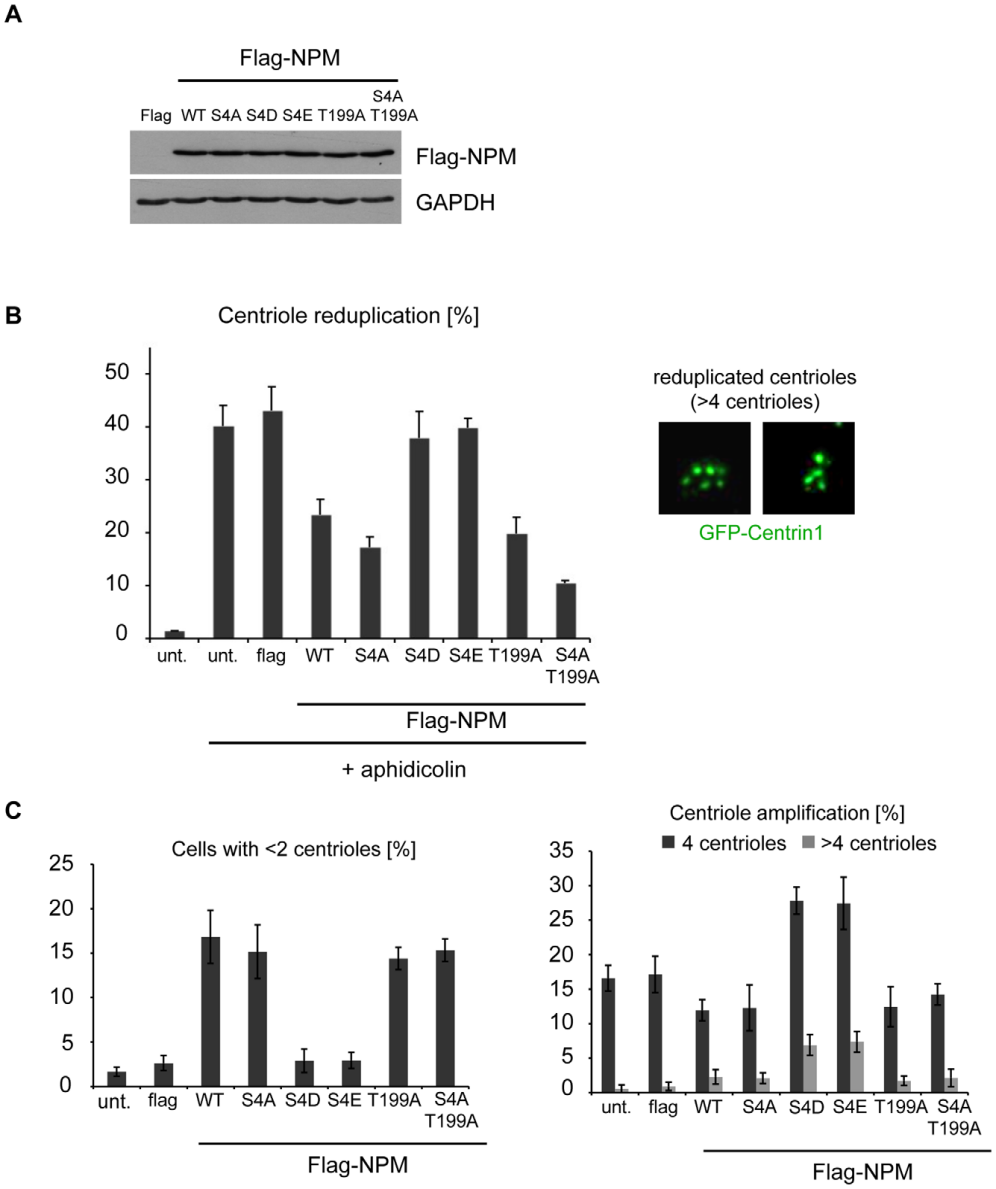
Serine 4 phosphorylated NPM/B23 promotes centrosome duplication. **A** After transfections with Flag-tagged NPM/B23 WT and non-phosphorylatable mutants (S4A, T199A, S4A/T199A) or phospho-mimicking mutants (S4D, S4E), centriole reduplication was performed in U2OS GFP-Centrin1 cells with 1.9 µg/ml aphidicolin for 75 h. Afterwards, cells were fixed for immunofluorescence and cells lysates were prepared for Western blot analysis using anti-Flag antibodies to show comparable expression levels of the transfected constructs. Loading control, GAPDH. **B** Quantification of the centriole reduplication phenotype of experiments described in (A). Cells were analyzed by immunofluorescence using anti-Flag antibodies to detect transfected cells. Flag-positive cells were analyzed for centriole reduplication (>4 GFP-Centrin1 dots/centrioles). For quantification, >150 transfected cells were counted in three independent experiments. Insets show representative centriole reduplication phenotypes. GFP-Centrin1, green. **C** Non-phosphorylatable or phospho-mimicking Flag-tagged NPM/B23 mutants described in (A) were overexpressed in U2OS GFP-Centrin1 cells for 48 h. After immunofluorescence using anti-Flag antibodies, Flag-positive cells were scored for centriole dilution (<2 GFP-Centrin1 dots/centrioles) or centriole amplification (4 or >4 GFP-Centrin1 dots/centrioles). Quantifications are means +/− standard deviation from three independent experiments, >150 transfected cells were counted for each experiment.

NPM/B23 has been shown to specifically associate with the unduplicated centrosome [11]. Upon Cdk2/Cyclin E-mediated phosphorylation on T199 it dissociates from the centrosome to allow centriole duplication [11] [12]. In addition, Plk2 also triggers centriole duplication [6] [7]. To examine the role of phosphorylation of NPM/B23 on S4 in centriole duplication, we generated in addition to the NPM/B23 S4A mutant also the NPM/B23 S4A/T199A double mutant and the phospho-mimicking mutants S4D (D, aspartic acid) and S4E (E, glutamic acid). Equal expression of the constructs is shown in Western blots in Fig. 6A. These constructs were then used in a centrosome duplication assay. This assay is based on the fact that in some cell lines, as for example U2OS, DNA replication can be experimentally uncoupled from the centrosome cycle in the presence of DNA synthesis inhibitors as HU or aphidicolin. This treatment allows multiple rounds of centriole duplication to occur in the absence of DNA replication or cytokinesis. In untreated cells, the vast majority of U2OS cells contained 2 or 4 centrioles depending on the cell cycle stage. In contrast, most aphidicolin-treated U2OS cells had multiple centrioles (>4) (Fig. 6B right) [23]. To perform this experiment we used a U2OS cell line that stably expressed GFP-Centrin1, a centriolar marker localizing at the distal lumen of the centrioles [24]. Results in Fig. 6B show that while NPM/B23 WT interferes with centrosome reduplication (25% of the cells exhibited the reduplication phenotype, i.e. >4 centrioles), this effect was even more pronounced when the S4A mutant was transfected (18% of cells with >4 centrioles). Interestingly, transfection of the double mutant S4A/T199A had the strongest effect with 11% of cells that contained >4 centrioles, which suggests that these two phosphorylations collaborate to promote centriole duplication. Together these results indicate that phosphorylation of NPM/B23 on S4 allows centriole reduplication.

The phospho-mimicking mutants NPM/B23 S4D and S4E did not show an effect on aphidicolin-induced centriole reduplication (Fig. 6B). To directly verify that a phosphorylation of NPM/B23 on S4 promotes centriole duplication, NPM/B23 mutants were overexpressed in unperturbed U2OS GFP-Centrin1 cells. Our findings shown in Fig. 6C (left panel) demonstrate again that expression of NPM/B23 WT and the mutants S4A and S4A/T199A led to an inhibition of centriole duplication resulting in a dilution of centrioles in these cells. Interestingly, overexpression of the phospho-mimicking mutants S4D or S4E did lead to an increase of centriole numbers (Fig. 6C, right panel). This demonstrates, that NPM/B23 in an unphosphorylated NPM/B23 S4 state inhibits centriole reduplication as well as unperturbed centriole duplication. In addition, we show that phosphorylation of NPM/B23 S4 directly promotes centriole duplication in unperturbed cells. Since NPM/B23 S4 phosphorylation in S-phase depends on Plk2 activity (Fig. 4 and 5), Plk2-mediated phosphorylation of NPM/B23 seems to trigger centriole duplication.

**Figure 7:**
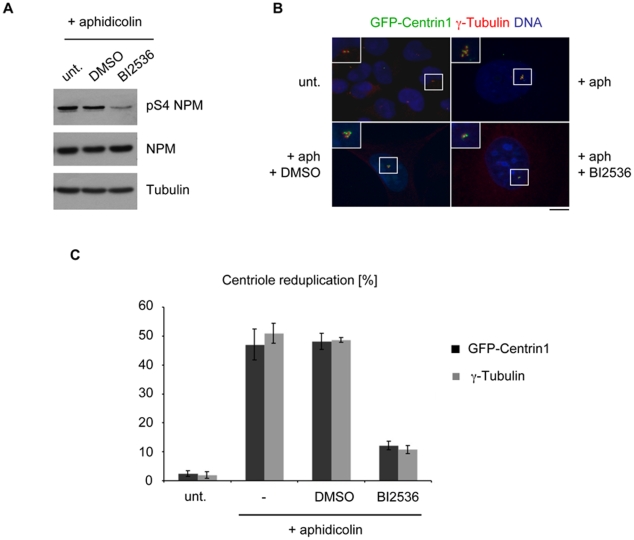
Treatment with BI2536, a Polo-kinase inhibitor, interferes with centriole reduplication. **A** U2OS GFP-Centrin1 cells were treated with 1.9 µg/ml aphidicolin to induce centriole reduplication for 75 h. In parallel, treatment with 100 nM BI2536 or DMSO as control was performed. Cell extracts were resolved by SDS-PAGE and analyzed on Western blot level with anti-NPM/B23 and anti-pS4 NPM/B23 antibodies. α-Tubulin, loading control. **B** Indirect immunofluorescence analysis from the experiment described in (A) was carried out specific for γ-Tubulin after methanol fixation. Insets show enlargement of centrioles. GFP-Centrin1, green; γ-Tubulin, red; DNA, blue. Scale bar, 10 µm. **C** For statistics, the experiment in (B) was also scored for centriole reduplication (>4 GFP-Centrin1 dots/centrioles or >2 γ-Tubulin dots/centrosomes) by microscopy. For quantification, more than 200 cells were analyzed for centriole reduplication in three independent experiments. Black bars, GFP-Centrin1 signal; grey bars, γ-Tubulin staining; unt, untreated; aph, aphidicolin.

**Figure 8:**
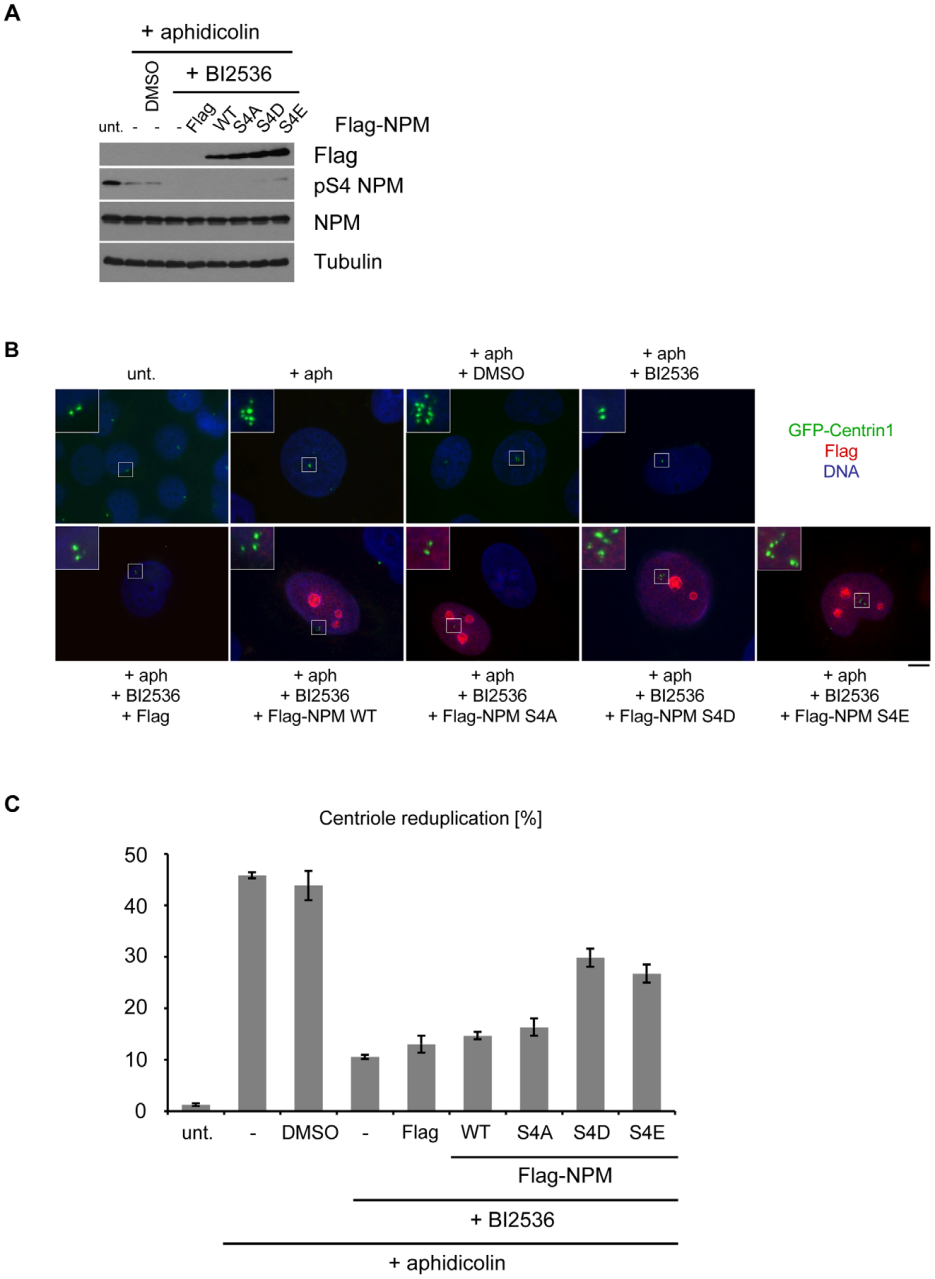
Overexpression of phospho-mimicking NPM/B23 S4D and S4E mutants rescues BI2536-inhibited centriole reduplication. **A** U2OS GFP-Centrin1 cells were transfected with Flag-NPM/B23 constructs for 8 h and then subjected to a centrosome duplication assay with 1.9 µg/ml aphidicolin for 75 h. At the same time, cells were incubated either with 100 mM BI2536 or DMSO as control. Western blot analysis showing the expression of all constructs and pS4 NPM/B23 protein levels. **B** After methanol fixation, indirect immunofluorescence analysis with anti-Flag antibodies was performed. Insets show enlargements of centrioles. GFP-Centrin1, green; Flag, red; DNA, blue. Scale bar, 10 µm. **C** For statistics, U2OS GFP-Centrin1 cells from indirect immunofluorescence analyses in (B) were scored for centriole reduplication (>4 GFP-Centrin1 dots/centrioles). In case of transfections, only Flag-positive cells were analyzed. In total, 150 cells were counted each in 3 independent experiments to calculate means +/− standard deviation. unt, untreated; aph, aphidicolin.

To further demonstrate that Plk2 regulates centriole duplication via NPM/B23 phosphorylation on S4 we again used a centrosome duplication assay as described in Fig. 6B. To verify our results, we applied the Plk inhibitor BI2536. In S-phase arrested cells, BI2536 only inhibits Plk2 since Plk1 is not active (Fig. 4B and 4C) and therefore cannot affect NPM/B23 S4 phosphorylation at this cell cycle stage (Fig. 5C). Treatment with the inhibitor reduced NPM/B23-S4 phosphorylation by leaving overall levels of NPM/B23 unaltered (Fig. 7A). Furthermore, the inhibitor also interferes with centriole reduplication since the reduplication phenotype (cells with >4 centrioles) exhibited by around 50% of the cells was reduced to 12% (Fig. 7B and 7C). Similar results were obtained when centrosomes were stained with γ-Tubulin. We then tested whether the effect of BI2536 on centriole reduplication from Fig. 7 could be reversed by the overexpression of NPM/B23 mutants used in Fig. 6. Fig. 8A demonstrates that U2OS GFP-Centrin1 cells that were transfected with NPM/B23 WT, S4A, S4D and S4E mutants and arrested with aphidicolin showed a decreased phosphorylation on S4 in the presence of the small molecule inhibitor BI2536. Expression of the NPM/B23 WT or the S4A mutant lead to reduced centrosome reduplication upon treatment with the inhibitor, resulting in inhibition of Plk2 activity, to an extent that was similar to control transfections. Importantly, however, when the phospho-mimicking mutants NPM/B23 S4D or S4E were transfected the reduplication phenotype could be restored up to 2-fold in comparison to controls suggesting that expression of these mutants can partially rescue the effects on centriole duplication induced by BI2536 (Fig. 8B and 8C). Since NPM/B23 WT had only a minimal effect, we anticipate that only a small amount of the very abundant NPM/B23 protein in the cells is indeed phosphorylated on S4.

**Figure 9:**
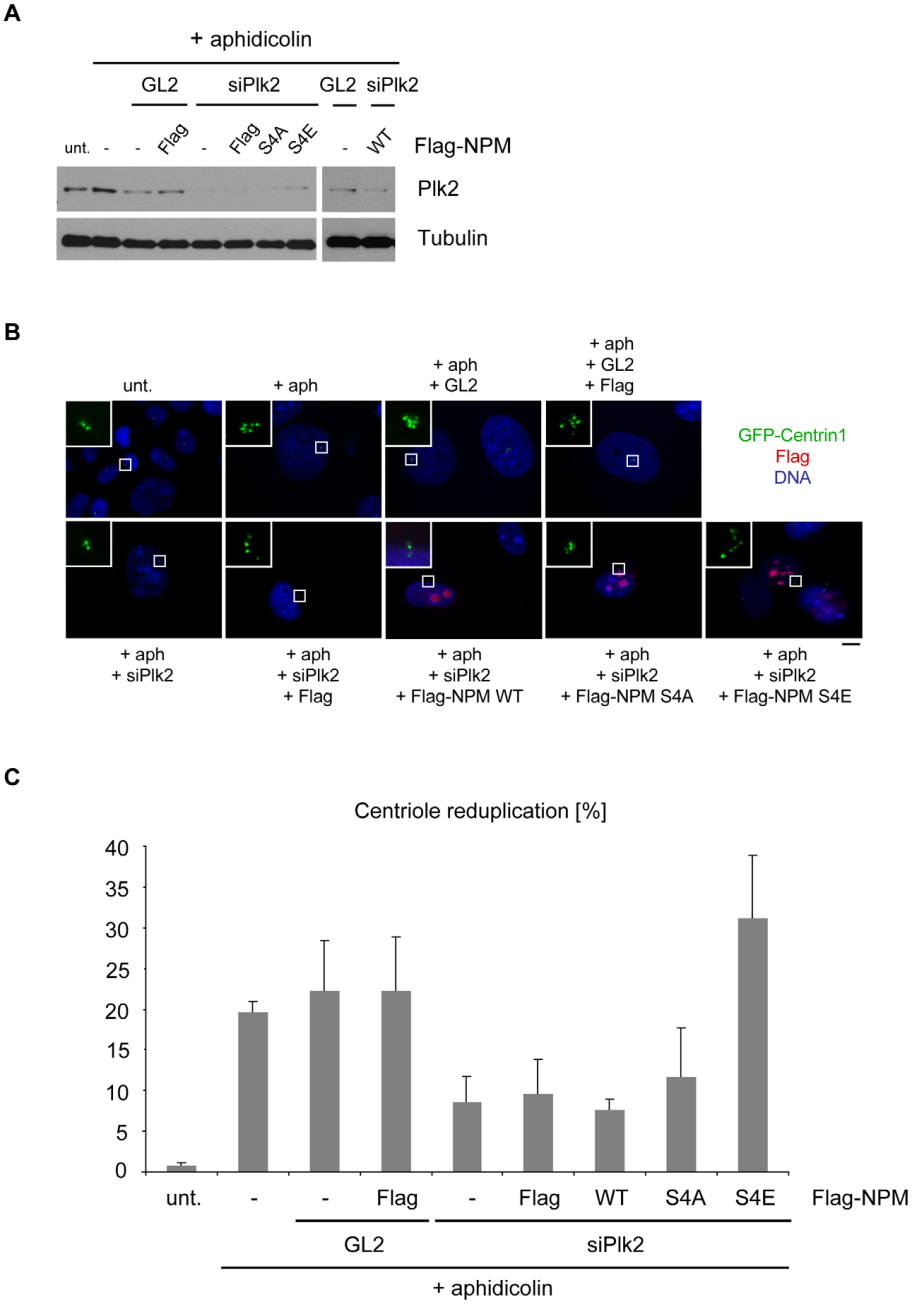
Overexpression of the phospho-mimicking NPM/B23 S4E mutant rescues inhibition of centriole reduplication induced by Plk2 RNAi. **A** U2OS GFP-Centrin1 cells were subjected to siRNA transfections (siPlk2 or GL2 as control) using Lipofectamine2000, transfected with Flag-NPM/B23 constructs for 8 h and then subjected to a centrosome duplication assay in the presence of 1.9 µg/ml aphidicolin for 75 h. Western blot analysis demonstrated the downregulation of Plk2 by specific antibodies. loading control, α-Tubulin **B** After fixing cells from experiment in (A) with methanol indirect immunofluorescence analyses were carried out with anti-Flag antibodies. Insets show enlargements of centrioles. GFP-Centrin1, green; Flag, red; DNA, blue. Scale bar, 10 µm. **C** For statistics, U2OS GFP-Centrin1 cells from indirect immunofluorescence analyses in (B) were scored for centriole reduplication (>4 GFP-Centrin1 dots/centrioles). In case of transfections, only Flag-positive cells were analyzed. In total, 200 cells were counted each in 3 independent experiments to calculate means +/− standard deviation. unt, untreated, aph, aphidicolin.

As BI2536 also inhibits Plk1 kinase activity and to strengthen the results obtained we used Plk2 siRNAs to specifically interfere with Plk2-dependent centriole duplication. Again U2OS GFP-Centrin1 cells were transfected with NPM/B23 WT, S4A and S4E mutants and arrested in S-phase by aphidicolin treatment. Down-regulation of Plk2 is shown in Fig. 9A. While either NPM/B23 WT or S4A transfections did not restore the Plk2 siRNA reduction of the reduplication phenotype, we found that expression of the S4E mutant restored the phenotype (Fig. 9B and 9C). Taken together, these results again show that Plk2-mediated phosphorylation of NPM/B23 on S4 in S-phase promotes centriole duplication.

## Discussion

In this report, we set out to search for novel binding partners and/or substrates of the kinase Plk2 in order to understand how Plk2 triggers centrosome duplication. We have identified NPM/B23 as a novel interacting protein of Plk2 and have shown that both endogenous and exogenous Plk2 bind to NPM/B23. Furthermore, we demonstrate that NPM/B23 is a substrate of Plk2 *in vivo* and that phosphorylation by Plk2 occurs on serine 4 in S-phase, which triggers centriole duplication.

NPM/B23 is a very abundant and highly conserved phosphoprotein that resides in the nucleoli, although it rapidly shuttles between the nucleus and the cytoplasm [15]. By shuttling between cellular compartments, NPM/B23 takes part in various cellular processes including maintenance of genomic stability. Its phosphorylation state is modified by a number of protein kinases during the cell cycle including casein kinase II [25] during interphase. In mitosis, the localization of NPM/B23 is regulated by kinases including Cdk1/Cyclin B [26], Nek2 [27] and Plk1 [14]. Before the cell enters mitosis, the interphase centrosome duplicates which has to take place in coordination with other cell cycle events including DNA synthesis. The process on how initiation of centriole duplication occurs and how it is regulated is not well understood. During S-phase, a new (daughter) centriole grows from the lateral surface of each pre-existing (mother) centriole, due to the combined influence of Cdk2/Cyclin E activity and a conserved set of centriole assembly factors [2] [28] [29]. In contrast to disengagement in mitosis, new centriole growth occurs in interphase and is dependent on Cdk2/Cyclin E [4]. NPM/B23 has been shown to associate to the unduplicated centrosome and dissociates from it upon phosphorylation on T199 by Cdk2/Cyclin E [11] [12]. A NPM/B23 T199A mutant specifically inhibits centrosome duplication [12]. Our own data demonstrate that expression of a NPM/B23 S4A mutant also interferes with centrosome duplication in both S-phase-arrested U2OS cells and in an unperturbed U2OS cell cycle (Fig. 6B and 6C). Importantly, we found that expression of NPM/B23 WT and S4A leads to a loss of centrioles in these cells. Interestingly, a NPM/B23 T199A/S4A double mutant further increased the effect suggesting that both phosphorylations need to occur to obtain a pronounced effect on centrosome duplication. This presumption is also supported by the fact that Plk2 and Cdk2/Cyclin E are activated at the same time in the cell cycle near the G1/S phase transition [6]. Plk1 can also phosphorylate NPM/B23 on S4 in mitosis [14]. In S-phase, Plk1 protein levels are low (Fig. 4A) since it becomes degraded after cytokinesis by APC/Cdh1 [30] and is consequently not active (Fig. 4B and 4C). Therefore, it is unlikely that Plk1 kinase activity accounts for the phosphorylation on S4 in S-phase extracts. In addition, a direct phosphorylation of NPM/B23 on S4 by Plk1 in S-phase could be excluded by RNAi experiments (Fig. 5C). Recent data suggest that Plk1 rather acts in coordination with separase to control centriole disengagement at mitotic exit, which is a prerequisite for centriole duplication to occur in S-phase [31]. Moreover, this work shows that Plk2 activity directly effects NPM/B23 S4 phosphorylation in S-phase and that a phosphorylation of NPM/B23 S4 promotes centriole duplication (Fig. 6C, left panel). These data are particular strengthened by experiments presented in Fig. 8 and 9 when we either used the small molecule inhibitor BI2536 or Plk2 specific siRNAs in S-phase arrested cells to ablate Plk2 function which leads to a reduction in the centriole reduplication phenotype. In both setups the phospho-mimicking mutant, S4E, could rescue the phenotype while the non-phosphorylatable mutant S4A did not. Taken together, our results demonstrate that centriole duplication in S-phase is positively regulated by a phosphorylation of NPM/B23 on S4 by Plk2, a S-phase linked Polo-like kinase.

Interestingly, Plk2−/− mice are viable [32]. We anticipate that there might be a functional redundancy between polo-like kinases in the early mouse development. Therefore, either Plk1 or Plk3, which has been recently found to be involved in S-phase entry, [33] could take over the function of Plk2 in centriole duplication.

Although Plk4 is a key trigger of centriole duplication [34] [35] we did not find that phosphorylation on NPM/B23 S4 was dependent on Plk4 *in vivo* (Fig. 5G). Cdk2/Cyclin E seems to collaborate with both Plk4 and Plk2 [7] [33]. It is therefore likely that the two Polo-like kinases possess different substrate specificities. This can be explained by their distinct structural properties in particular the different Polo-boxes. Their different architectures argue against a conservation of phosphoprotein binding function since residues that are most involved in phosphopeptide binding by Plk1 or Plk2 are not conserved in Plk4 [8] [9].

In conclusion, Plk2 may contribute to aneuploidy and tumorigenesis by uncontrolled phosphorylation of NPM/B23 on S4 at the G1/S phase transition and thereby triggering centriole reduplication. In fact, phosphorylation of NPM/B23 is increased in several tumor cell lines including melanoma cell lines [36] [37], and is used as a marker of melanoma progression and aneuploidy [37]. Thus phosphorylation of NPM/B23 on S4 by Plk2 provides a novel mechanism that links centrosome duplication to chromosomal instability and tumorigenesis.

## Materials and Methods

### Cell Culture, Synchronization and Transfection

HeLa, 293T, U2OS and U2OS GFP-Centrin1 (S.Duensing, Pittsburgh) cells were cultured in DMEM (Sigma) containing 10% fetal bovine serum (PPA), 5% of 200 mM L-glutamine (Invitrogen) and if necessary 5% penicillin-streptomycin solution (Invitrogen) at 37°C in 5% CO_2_. Cells were synchronized in S-phase with 4 mM hydroxyurea (Sigma) for 40 h. To arrest cells in metaphase, they were treated with 100 ng/ml nocodazole (Sigma) for 15 h. Inhibition of Polo-kinase activity was achieved by a 2 h incubation with 100 nM BI2536 (Boehringer Ingelheim). 293T cells were transfected using calcium phosphate and HBS buffer (10 mM HEPES pH 7.4, 140 mM NaCl, 3 mM EDTA). U2OS GFP-Centrin1 cells were transfected with Lipofectamine2000 (Invitrogen) according to the manufacturers instructions for DNA plasmids. SiRNA transfections and vector-mediated RNAi were performed with Lipofectamine2000 (Invitrogen) as described previously [6] [7] [15] [38].

### Recombinant protein production

Expression of recombinant proteins was carried out in *E.coli* BL21 cells. After collecting bacterial cells, they were lysed in 50 mM Tris-HCl pH 7.5, 250 mM NaCl, 2 mM MgCl_2_, 4 mM β-mercaptoethanol, 5% glycerol, 0.4 mg/ml lysozyme (Sigma) for 30 min on ice and subjected to sonification. Following centrifugation, the supernatant was incubated with glutathione agarose (Sigma-Fluka) for GST-tagged proteins, or Ni-NTA agarose (Biorad) for His-tagged proteins for 3 h at 4°C. After washing the agarose three times, elution of proteins was performed with either 20 mM reduced L-glutathione (Sigma) for GST-tagged protein or with 300 mM imidazole (Sigma) for His-tagged proteins.

### Mass spectrometry analysis

EGFP-Plk2 immunoprecipitates were resolved by SDS-polyacrylamid gel electrophoresis (PAGE) and co-immunoprecipitating proteins were detected in gel by silver staining. In-gel digestion and mass spectrometric analysis was performed as described previously [39]. In brief, protein bands of interest were cut out and digested with trypsin after reduction and alkylation of cystines. Peptides were separated by nanoHPLC and analyzed with an on-line coupled ESI QTOF MS (Applied Biosystems). Only doubly and triply charged ions were selected for fragmentation. The uninterpreted MS/MS spectra were searched against the NCBInr database, taxonomy human (191517 sequences, download 16.03.2007) using the Mascot software (Matrix Science). The algorithm was set to use trypsin as enzyme, allowing at maximun for one missed cleavage site and assuming carbamidomethyl as a fixed modification of cysteine, and oxidized methionine and deamidation of asparagines and glutamines as variable modifications. Mass tolerance was set to 0.1 Da for MS and MS/MS, respectively. A significant threshold of p<0.05 has been used to distinguish between correct and false peptide identifications.

### Antibodies

anti-FlagM2, anti-α-Tubulin; anti-γ-Tubulin [Sigma]; anti-Myc (9E10), anti-Plk1 (F-8), anti-CyclinE (H-12), anti-GST [SantaCruz], anti-NPM/B23 [Zymed]; anti-GAPDH, anti-GFP [Abcam]; anti-pS4 NPM/B23, anti-pT199 NPM/B23 [Cell Signaling Technology]; anti-NPM/B23 [M.Schmidt-Zachmann, DKFZ, Heidelberg], anti-Plk2 CDL-21 [6], anti-Cyclin B1 [40], anti-Plk4 [7].

### Immunoprecipitations, Western blot analysis and Far Western blot analysis

Cell extracts were prepared with lysis buffer (40 mM Tris-HCl pH 7.5, 0.5% NP-40, 150 mM NaCl, 5 mM EDTA, 10 mM β-glycerolphosphate, 5 mM NaF and protease inhibitor cocktail). For immunoprecipitation, 3–5 mg cell extract were incubated with 3–5 µg primary antibodies either for 2 h or for overnight at 4°C. 10 µl of seated Protein G sepharose (GE Healthcare) was added and reactions were incubated for 1 h at 4°C. Immunocomplexes were washed three times with lysis buffer and examined by Western blot analysis. For Western blot analysis a standard protocol was used [41] [42]. Immunoreactive signals were detected with ECL Western blotting substrate (Thermo Scientific) or Super Signal West Dura Extended Duration Signal (Thermo Scientific). Far Western blot analysis was carried out using 250 ng of His-tagged NPM/B23 as bait and ligand binding was performed in 5% milk powder in TBST (40 mM Tris-HCl pH7.5, 300 mM NaCl, 0.2% Tween20), with 1 µg/ml GST-Plk2-PBD, GST-Plk2-PBD mutant or GST alone for 6 h at 4°C. Bound protein was then detected by Western blot analysis using anti-GST antibodies.

### Pulldown and *In vitro* binding assay

Zz-tagged proteins [43] were bound to IgG sepharose (GE Healthcare) in 50 mM Tris-HCl pH 7.5, 150 mM NaCl, 5 mM MgCl_2_, 5% glycerol, 10 mM β-glycerolphosphate, 5 mM NaF and protease inhibitor cocktail. IgG sepharose was washed three times and 10 mg HeLa extract was added followed by incubation for 3 h at 4°C. After washing the sepharose beads again, bound proteins were eluted with 200 mM NaCl followed by 1 M NaCl and examined by Western blot analysis. For *in vitro* binding assays either 25 µg GST-NPM/B23 bound to glutathione agarose or immunoprecipitated Flag-Plk2 fragments bound to Protein G sepharose were incubated with 8 µl *in vitro* translated HA-Plk2 (TnT Reticulocyte Kit, Promega) and 15 µg recombinant His-NPM/B23, respectively, in PBS containing 0.05% NP-40 for 2 h at 4°C. After washing complexes three times with PBS containing 0.05% NP-40, protein complexes were separated by SDS-PAGE. Afterwards, radioactively labeled proteins were detected after Coomassie blue staining by autoradiography while non-radioactive binding assays were analyzed by Western blot analysis.

### *In vitro* kinase assay

To assay Plk1 and Plk2 kinase activities, immunoprecipitated or bacterially expressed and purified kinases were incubated either with immunoprecipitated proteins or 500 ng recombinant proteins from bacteria as substrates and analyzed as described previously [6]. Non-radioactive kinase assays were examined by Western blot analysis using anti-pS4 NPM/B23 antibodies.

### Centrosome duplication assay

After DNA transfections for 8 h, U2OS GFP-Centrin1 cells were treated with 1.9 µg/ml aphidicolin (Sigma) for 75 h. Polo-kinase inhibition by 100 nM BI2536 was carried out during aphidicolin treatment. Plk2 RNAi was performed as described in [7], after 20 h DNA transfections followed. Cells were then subjected to immunofluorescence analysis.

### Immunofluorescence microscopy

U2OS or U2OS GFP-Centrin1 cells on acid-washed coverslips were fixed with 100% icecold methanol for 6 min at −20°C. After blocking in 3% bovine serum albumin (Sigma) in PBS for 30 min, primary antibody incubation (anti-FlagM2, anti-γ-Tubulin [Sigma]; anti-Plk2 CDL-21 [6]; anti-GT335 [Carsten Janke, CRBM, Montpellier]) was carried out for 1 h at room temperature, followed by a 30 min incubation with an secondary antibody (anti-mouse Alexa594, anti-rabbit Alexa488 [Invitrogen]). DNA was stained by Hoechst 33342 (Molecular probes). For imaging, the Leica Leitz DMRBE microscope equipped with a Leica FWII camera was used. Image processing was performed by ImageJ (NIH).

## References

[1] Doxsey S, McCollum D, Theurkauf W (2005). Centrosomes in cellular regulation.. Annu Rev Cell Dev Biol.

[2] Nigg EA (2007). Centrosome duplication: of rules and licenses.. Trends Cell Biol.

[3] Ganem NJ, Godinho SA, Pellman D (2009). A mechanism linking extra centrosomes to chromosomal instability.. Nature.

[4] Tsou MF, Stearns T (2006). Mechanism limiting centrosome duplication to once per cell cycle.. Nature.

[5] Kleylein-Sohn J, Westendorf J, Le Clech M, Habedanck R, Stierhof YD (2007). Plk4-induced centriole biogenesis in human cells.. Dev Cell.

[6] Warnke S, Kemmler S, Hames RS, Tsai HL, Hoffmann-Rohrer U (2004). Polo-like kinase-2 is required for centriole duplication in mammalian cells.. Curr Biol.

[7] Cizmecioglu O, Warnke S, Arnold M, Duensing S, Hoffmann I (2008). Plk2 regulated centriole duplication is dependent on its localization to the centrioles and a functional polo-box domain.. Cell Cycle.

[8] Leung GC, Hudson JW, Kozarova A, Davidson A, Dennis JW (2002). The Sak polo-box comprises a structural domain sufficient for mitotic subcellular localization.. Nat Struct Biol.

[9] Elia AE, Rellos P, Haire LF, Chao JW, Ivins FJ (2003). The molecular basis for phosphodependent substrate targeting and regulation of Plks by the Polo-box domain.. Cell.

[10] Hinchcliffe EH, Sluder G (2002). Two for two: Cdk2 and its role in centrosome doubling.. Oncogene.

[11] Okuda M, Horn HF, Tarapore P, Tokuyama Y, Smulian AG (2000). Nucleophosmin/B23 is a target of CDK2/cyclin E in centrosome duplication.. Cell.

[12] Tokuyama Y, Horn HF, Kawamura K, Tarapore P, Fukasawa K (2001). Specific phosphorylation of nucleophosmin on Thr(199) by cyclin-dependent kinase 2-cyclin E and its role in centrosome duplication.. J Biol Chem.

[13] Zatsepina OV, Rousselet A, Chan PK, Olson MO, Jordan EG (1999). The nucleolar phosphoprotein B23 redistributes in part to the spindle poles during mitosis.. J Cell Sci.

[14] Zhang H, Shi X, Paddon H, Hampong M, Dai W (2004). B23/nucleophosmin serine 4 phosphorylation mediates mitotic functions of polo-like kinase 1.. J Biol Chem.

[15] Wang W, Budhu A, Forgues M, Wang XW (2005). Temporal and spatial control of nucleophosmin by the Ran-Crm1 complex in centrosome duplication.. Nat Cell Biol.

[16] Grisendi S, Mecucci C, Falini B, Pandolfi PP (2006). Nucleophosmin and cancer.. Nat Rev Cancer.

[17] Grisendi S, Bernardi R, Rossi M, Cheng K, Khandker L (2005). Role of nucleophosmin in embryonic development and tumorigenesis.. Nature.

[18] Lowery DW, Mohammad DH, Elia AE, Yaffe MB (2004). The Polo-box domain: a molecular integrator of mitotic kinase cascades and Polo-like kinase function.. Cell Cycle.

[19] Kang YH, Park JE, Yu LR, Soung NK, Yun SM (2006). Self-regulated Plk1 recruitment to kinetochores by the Plk1-PBIP1 interaction is critical for proper chromosome segregation.. Mol Cell.

[20] Johnson EF, Stewart KD, Woods KW, Giranda VL, Luo Y (2007). Pharmacological and functional comparison of the polo-like kinase family: insight into inhibitor and substrate specificity.. Biochemistry.

[21] Golsteyn RM, Mundt KE, Fry AM, Nigg EA (1995). Cell cycle regulation of the activity and subcellular localization of Plk1, a human protein kinase implicated in mitotic spindle function.. J Cell Biol.

[22] Steegmaier M, Hoffmann M, Baum A, Lenart P, Petronczki M (2007). BI 2536, a potent and selective inhibitor of polo-like kinase 1, inhibits tumor growth in vivo.. Curr Biol.

[23] Meraldi P, Lukas J, Fry AM, Bartek J, Nigg EA (1999). Centrosome duplication in mammalian somatic cells requires E2F and Cdk2-cyclin A.. Nat Cell Biol.

[24] Paoletti A, Moudjou M, Paintrand M, Salisbury JL, Bornens M (1996). Most of centrin in animal cells is not centrosome-associated and centrosomal centrin is confined to the distal lumen of centrioles.. J Cell Sci.

[25] Chan PK, Liu QR, Durban E (1990). The major phosphorylation site of nucleophosmin (B23) is phosphorylated by a nuclear kinase II.. Biochem J.

[26] Peter M, Nakagawa J, Doree M, Labbe JC, Nigg EA (1990). Identification of major nucleolar proteins as candidate mitotic substrates of cdc2 kinase.. Cell.

[27] Yao J, Fu C, Ding X, Guo Z, Zenreski A (2004). Nek2A kinase regulates the localization of numatrin to centrosome in mitosis.. FEBS Lett.

[28] Azimzadeh J, Bornens M (2007). Structure and duplication of the centrosome.. J Cell Sci.

[29] Bettencourt-Dias M, Glover DM (2007). Centrosome biogenesis and function: centrosomics brings new understanding.. Nat Rev Mol Cell Biol.

[30] Lindon C, Pines J (2004). Ordered proteolysis in anaphase inactivates Plk1 to contribute to proper mitotic exit in human cells.. J Cell Biol.

[31] Tsou MF, Wang WJ, George KA, Uryu K, Stearns T (2009). Polo kinase and separase regulate the mitotic licensing of centriole duplication in human cells.. Dev Cell.

[32] Ma S, Charron J, Erikson RL (2003). Role of Plk2 (Snk) in mouse development and cell proliferation.. Mol Cell Biol.

[33] Zimmerman WC, Erikson RL (2007). Polo-like kinase 3 is required for entry into S phase.. Proc Natl Acad Sci U S A.

[34] Bettencourt-Dias M, Rodrigues-Martins A, Carpenter L, Riparbelli M, Lehmann L (2005). SAK/PLK4 Is Required for Centriole Duplication and Flagella Development.. Curr Biol.

[35] Habedanck R, Stierhof YD, Wilkinson CJ, Nigg EA (2005). The Polo kinase Plk4 functions in centriole duplication.. Nat Cell Biol.

[36] Nagano K, Shinkawa T, Mutoh H, Kondoh O, Morimoto S (2009). Phosphoproteomic analysis of distinct tumor cell lines in response to nocodazole treatment.. Proteomics.

[37] Bernard K, Litman E, Fitzpatrick JL, Shellman YG, Argast G (2003). Functional proteomic analysis of melanoma progression.. Cancer Res.

[38] Stucke VM, Baumann C, Nigg EA (2004). Kinetochore localization and microtubule interaction of the human spindle checkpoint kinase Mps1.. Chromosoma.

[39] Tegha-Dunghu J, Neumann B, Reber S, Krause R, Erfle H (2008). EML3 is a nuclear microtubule-binding protein required for the correct alignment of chromosomes in metaphase.. J Cell Sci.

[40] Hoffmann I, Clarke PR, Marcote MJ, Karsenti E, Draetta G (1993). Phosphorylation and activation of human cdc25-C by cdc2–cyclin B and its involvement in the self-amplification of MPF at mitosis.. Embo J.

[41] Blomberg I, Hoffmann I (1999). Ectopic expression of Cdc25A accelerates the G(1)/S transition and leads to premature activation of cyclin E- and cyclin A-dependent kinases.. Mol Cell Biol.

[42] Hassepass I, Voit R, Hoffmann I (2003). Phosphorylation at serine 75 is required for UV-mediated degradation of human Cdc25A phosphatase at the S-phase checkpoint.. J Biol Chem.

[43] Gorlich D, Henklein P, Laskey RA, Hartmann E (1996). A 41 amino acid motif in importin-alpha confers binding to importin-beta and hence transit into the nucleus.. Embo J.

